# Important advances in Alzheimer’s disease from the use of induced pluripotent stem cells

**DOI:** 10.1186/s12929-019-0501-5

**Published:** 2019-02-06

**Authors:** Fernanda Majolo, Daniel Rodrigo Marinowic, Denise Cantarelli Machado, Jaderson Costa Da Costa

**Affiliations:** Brain Institute of Rio Grande do Sul (BraIns), Postgraduate Program in Medicine and Health Sciences (PUCRS), Pontifical Catholic University of Rio Grande do Sul, Porto Alegre, RS 90610000 Brazil

**Keywords:** Alzheimer’s disease, Induced pluripotent stem cells, PubMed

## Abstract

Among the various types of dementia, Alzheimer’s disease (AD) is the most prevalent and is clinically defined as the appearance of progressive deficits in cognition and memory. Considering that AD is a central nervous system disease, getting tissue from the patient to study the disease before death is challenging. The discovery of the technique called induced pluripotent stem cells (iPSCs) allows to reprogram the patient’s somatic cells to a pluripotent state by the forced expression of a defined set of transcription factors. Many studies have shown promising results and made important conclusions beyond AD using iPSCs approach. Due to the accumulating knowledge related to this topic and the important advances obtained until now, we review, using PubMed, and present an update of all publications related to AD from the use of iPSCs. The first iPSCs generated for AD were carried out in 2011 by Yahata et al. (PLoS One 6:e25788, 2011) and Yaqi et al. (Hum Mol Genet 20:4530–9, 2011). Like other authors, both authors used iPSCs as a pre-clinical tool for screening therapeutic compounds. This approach is also essential to model AD, testing early toxicity and efficacy, and developing a platform for drug development. Considering that the iPSCs technique is relatively recent, we can consider that the AD field received valuable contributions from iPSCs models, contributing to our understanding and the treatment of this devastating disorder.

## Introduction

Along with the aging population and as the main consequence of this, there is an increase in neurodegenerative diseases, including Alzheimer’s disease (AD) [137]. Dementia associated with several fatal clinical disorders is a considerable social, economic, and medical challenge [30]. By reaching approximately 50 million people, it has become a public health problem, with the global cost of US $818 billion for the treatment [3, 30]. Among the various types of dementia, AD is the most prevalent one and has been clinically defined as the appearance of progressive deficits in cognition and memory [10, 34].

There are two types of AD: Familial AD (FAD) and Sporadic AD (SAD). Both share clinical and pathological similarities, exhibiting progressive cognitive dementia, senile plaques consisting of amyloid β (Aβ) peptide and neurofibrillary tangles (NFTs) consisting of phosphorylated tau protein [62, 137]. Axonal transport defects, synapse loss and selective neuronal death are others cellular phenotypes shared by FAD and SAD [38, 43, 137].

FAD: early-onset, accounts for 5% of cases and is caused by highly penetrant and rare autosomal mutations of the PS1, PS2 and, less frequently, amyloid precursor protein (APP) genes. APP protein is fundamental for central nervous system (CNS) function acting in the formation of synapses, neurogenesis, axonal transport, signaling and plasticity [17, 41, 43, 58, 137].

SAD: late-onset, has established risk factors beyond age including cardiovascular disease, low education, depression, and the apolipoprotein-E4 (ApoE4) gene [30]. There are no clear dominant or recessive SAD mutations; however, many genetic variants have been identified and there is clearly a strong heritable component to the disorder [6, 137]. Thus, SAD has multifactorial origins, driven in part by a complex genetic profile and in part by environmental factors and the interaction of the two [30].

AD reaches the central nervous system (CNS); it is difficult to obtain samples of the patient’s nervous tissue before his death to study the disease [137]. It is possible, using the relatively recent technique called induced pluripotent stem cells (iPSCs), to study the genesis of diseases and identify new molecular targets that recapitulate the genetic background of the individual from disease models in the laboratory.

## Induced pluripotent stem cells

The models of diseases, truly representing real human diseases and their physiological peculiarities, that can be recreated in the laboratory, are needed to increase the success rate of new drug discoveries and developments [141]. In addition, the studies conducted in animal models do not efficiently show the translation of the therapeutic discovery for human use, although they are valuable in elucidating diseases and directing markers and genes associated with certain pathologies [27]. Specifically, regarding AD, vertebrate and nonvertebrate models can cause abnormal phenotypes mainly because of considerable overexpression of proteins. Notably, the mutations introduced into the endogenous mouse genes, unfortunately, do not recapitulate all the pathologies of the human AD [29, 137]. In addition, the studies already using postmortem tissue show major structural changes in the brain, both at the cellular and molecular levels.

After the discovery of the iPSCs in 2006 by Yamanaka and colleagues, it became possible to reprogram the patient’s somatic cells back to a pluripotent state, forcing the expression of a defined set of transcription factors. For this reprogramming, four transcription factors need to be introduced into fibroblasts through retroviruses. Consequently, the cells acquire a pluripotent stage with characteristics extremely similar to the embryonic stem cells [83]. The first transfection was performed on mouse fibroblasts [121], followed by transfection into human fibroblasts [120].

Considering the difficulty of obtaining CNS tissue from the patients with AD, the discovery of iPSCs shows a great potential and advantage to enable the modeling of in vitro diseases. For example, disease-specific cells from patients with AD can be produced with disorders without a clear pattern of inheritance and sporadic cases can be used in drug discovery programs [83].

Parkinson’s disease [78, 87, 101, 115], amyotrophic lateral sclerosis [25, 74], smooth muscle atrophy [31], and family dysautonomia [61] were the diseases initially studied using the iPSCs approach to model neurological diseases. These are monogenic disorders or versions of complex diseases caused by known mutations [72, 137].

### Important advances in Alzheimer’s disease using iPSCs

**Fig. 1:**
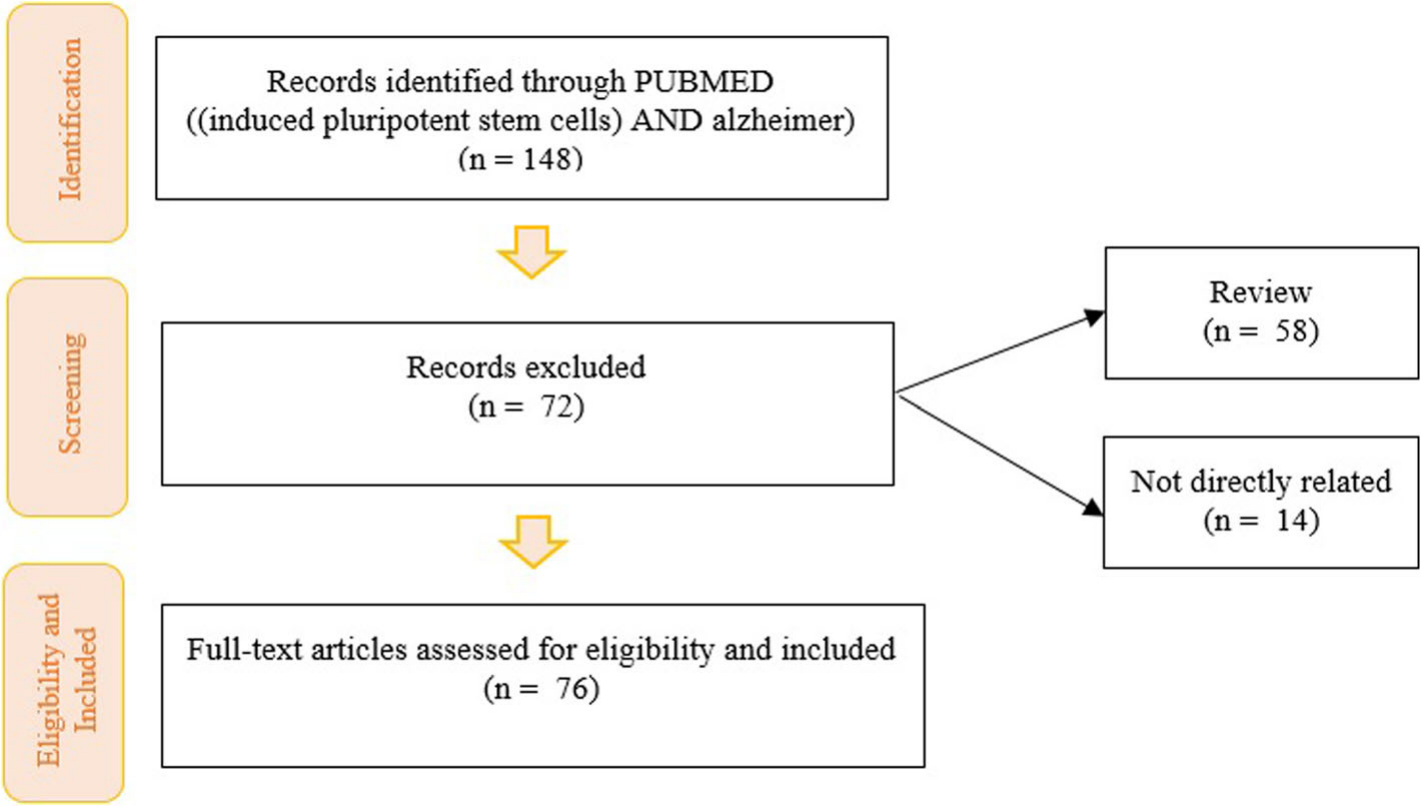
Flow diagram with the terms searched in the search engine

Many studies have shown promising results and important conclusions, beyond AD, using iPSCs allowing a better understanding of cellular and molecular targets. Here we review and present an update of all publications related to AD from the use of iPSCs (Table 1). Electronic databases, including PubMed, were searched for articles related to the use of iPSCs in AD research. Only full-text English-language articles were included. If the abstract met the inclusion criteria, the full-text article was obtained and reviewed. The flow diagram below shows which terms were searched and how many articles were excluded at each step and the reasons (Fig. 1).

**Table 1:** Update of all publications until now involving iPSCs approach in Alzheimer’s disease

TARGET		MAJOR FINDINGS	YEAR	REFERENCE
FAMILIAL AD	Aβ; Astrocyte; Lipoprotein receptor	APP-KO astrocytes have reduced cholesterol and elevated levels of sterol regulatory element-binding protein (SREBP) target gene transcripts and proteins, which were both downstream consequences of reduced lipoprotein endocytosis.	2018	Fong et al [36]
	Sendai-virus	Dermal fibroblasts of the patient were obtained and a line of iPSCs was successfully generated using the Sendai virus (SeV) delivery system.	2017	Wang et al [126]
	Aβ; Tau; Amyloid; Disease modeling; Selective vulnerability	Both the generation of Aβ and the responsiveness of TAU to Aβ are affected by neuronal cell type, with rostral neurons being more sensitive than caudal neurons. Cell-autonomous factors may in part dictate the pattern of selective regional vulnerability in human neurons in AD.	2017	Muratore et al [77]
	Aβ; sAPPα; Microengraving	The authors have uncovered the dynamic range of secretion profiles of analytes from single iPSCs-derived neuronal and glial cells and have molecularly characterized subpopulations of these cells through immunostaining and gene expression analyses.	2016	Liao et al [67]
	Aβ; Proteolytic Enzyme; BACE1	Demonstrate that A673T, a protective allele of APP, reproducibly reduces amyloidogenic processing of APP and also mildly decreases Aβ aggregation. These effects could together have an additive or even synergistic impact on the risk of developing AD.	2014	Maloney et al [71]
	Disease model	Over differentiation time to mature neuronal fates, APP expression and levels of Aβ increase dramatically. In both immature and mature neuronal fates, the APPV717I mutation affects both β- and γ-secretase cleavage of APP. β-secretase cleavage of APP is elevated leading to generation of increased levels of both APPsβ and Aβ. This mutation alters the initial cleavage site of γ-secretase, resulting in an increased generation of both Aβ42 and Aβ38. An increase in levels of total and phosphorylated Tau is observed in neurons with the APPV717I mutation. Treatment with Aβ-specific antibodies early in culture reverses the phenotype of increased total Tau levels, implicating altered Aβ production in FAD neurons in this phenotype.	2014	Muratore et al [76]
	5XFAD mice; Oligodendrocyte; Protein-iPSC; Proteomic analysis	Protein-iPSCs differentiated into glial cells and decreased plaque depositions in the 5XFAD transgenic AD mouse model. Transplanted protein-iPSCs mitigated the cognitive dysfunction observed in these mice. Proteomic analysis revealed that oligodendrocyte-related genes were upregulated in brains injected with protein-iPSCs, providing new insights into the potential function of protein-iPSCs.	2017	Cha et al [18]
	Endocytosis; Transcytosis	Accumulation of β-CTFs of APP, but not Aβ, slow vesicle formation from an endocytic recycling compartment marked by the transcytotic GTPase Rab11. The authors confirm previous results that endocytosis is affected in AD and extend these to uncover a neuron-specific defect. Decreased lipoprotein endocytosis and transcytosis to the axon suggest that a neuron-specific impairment in endocytic axonal delivery of lipoproteins and other key materials might compromise synaptic maintenance in FAD.	2016	Woodruff et al [128]
	Mono- and bi-allelic sequence changes	The authors generated human iPSCs with heterozygous and homozygous dominant early onset AD causing mutations in APP(Swe) and PS1(M146 V) and derived cortical neurons, which displayed genotype-dependent disease-associated phenotypes.	2016	Paquet et al [86]
	Therapeutic potential; Macrophage-like cells	In vitro, expression of NEP2 but not anti-Aβ scFv enhanced the effect to reduce the level of soluble Aβ oligomer in the culture medium and to alleviate the neurotoxicity of Aβ. The authors observed significant reduction in the level of Aβ in the brain interstitial fluid following administration of iPSCs-ML/NEP2.	2014	Takamatsu et al [122]
	ASM; Lysosomal depletion	Reveal a novel mechanism of ASM pathogenesis in AD that leads to defective autophagy due to impaired lysosomal biogenesis and suggests that partial ASM inhibition is a potential new therapeutic intervention for the disease.	2014	Lee et al [60]
	Apoptosis; NPCs	Premature neuronal differentiation with decreased proliferation and increased apoptosis occurred in AD-iPSCs-derived-NPCs once neuronal differentiation was initiated, together with higher levels of Aβ42 and phosphorylated tau.	2017	Yang et al [135]
	L282F mutation in PS1	The authors transfected skin fibroblasts with episomal iPSCs reprogramming vectors targeting human OCT4, SOX2, L-MYC, KLF4, NANOG, LIN28, and short hairpin RNA against TP53.	2016	Poon et al [89]
	L150P mutation in PS1	This gene-corrected line, L150P-GC-hiPSCs, serves as an isogenic control to the mutant line for future investigation of mechanisms and cellular phenotypes altered by this specific PS1 mutation.	2016	Poon et al [90]
	Disease model; M146I mutation	M146I-iPSCs were free of genomically integrated reprogramming genes, had the specific mutation but no additional genomic aberrancies, expressed the expected pluripotency markers and displayed in vitro differentiation potential to the three germ layers. The reported M146I-iPSCs line may be a useful resource for in vitro modeling of FAD.	2016	Li et al [64]
	Disease model; A79V mutation	A79V-iPSCs were free of genomically integrated reprogramming genes, had the specific mutation but no additional genomic aberrancies, expressed the expected pluripotency markers and displayed in vitro differentiation potential to the three germ layers.	2016	Li et al [65]
	L150P mutation	The iPSCs were established by co-electroporation with episomal plasmids containing hOCT4, hSOX2, hL-MYC, hKLF4, hNANOG, hLIN28, and short hairpin RNA against TP53. The iPSCs contained the specific heterozygous mutation c.449C > T, had normal karyotype, expressed the expected pluripotency genes and displayed in vitro differentiation potential to the three germ layers.	2016	Tubsuwan et al [123]
	3 different mutations; GSM	Biomarker signatures obtained with such models are misleading and that human neurons derived from hiPSCs provide a unique signature that will more accurately reflect drug response in human patients and in cerebrospinal fluid biomarker changes observed during GSM treatment.	2014	Liu et al [68]
	Non integrating vectors	Neurons from mutant hiPSC lines express PS1-A246E mutations themselves and show AD-like biochemical features, that is, amyloidogenic processing of APP indicated by an increase in Aβ42/Aβ40 ratio.	2014	Mahairaki et al [70]
	NPCs	PS1 mutant fibroblasts and NPCs produced greater ratios of Aβ42 to Aβ40 relative to their control counterparts, with the elevated ratio even more apparent in PS1 NPCs than in fibroblasts.	2014	Sproul et al [116]
	Allelic series mutations;	FAD PS1 mutations do not act as simple loss of PS1 function but instead dominantly gain an activity toxic to some, but not all, PS1 functions.	2013	Woodruff et al [129]
	Proteolytic APP processing	The human NSC-derived neurons express the neuron-specific APP(695) splice variant, BACE1, and all members of the γ-secretase complex. They also exhibit a differentiation-dependent increase in Aβ secretion and respond to the pharmacotherapeutic modulation by anti-amyloidogenic compounds, such as γ-secretase inhibitors and nonsteroidal anti-inflammatory drugs.	2012	Koch et al [54]
	Aβ42 secretion	FAD-iPSCs-derived differentiated neurons have increased toxic Aβ42 secretion, recapitulating the molecular pathogenesis of mutant presenilins. Secretion of Aβ42 from these neurons sharply responds to γ secretase inhibitors and modulators, indicating the potential for identification and validation of candidate drugs.	2012	Yagi et al [130]
	Amyloid; Bodily secretions; Cognitive impairment	FAD-iPSCs-derived differentiated neurons have increased Aβ 42 secretion, recapitulating the molecular pathogenesis of mutant presenilins.	2011	Yagi et al [131]
	BFCN; BFCNs; Electrophysiology	Cell lines harboring the PS2 N141I mutation displayed an increase in the Aβ42/40 in iPSCs-derived BFCNs. Neurons derived from PS2 N141I lines generated fewer maximum number of spikes in response to a square depolarizing current injection.	2017	Ortiz-Virumbrales et al [84]
	Fibroblast library; DIAN	The authors reprogrammed a subset of the DIAN fibroblast lines into iPSCs lines.	2018	Karch et al [52]
	Aβ hypothesis; Anti-cancer drugs; Clinical trials; Semagacestat; γ-by products; γ-secretase inhibitors	Some semagacestat effects are clearly different from a phenotype caused by a loss of function of presenilins, core proteins in the γ-secretase complex. Semagacestat increases intracellular byproduct peptides, produced along with Aβ through serial γ-cleavage of βAPP, as well as intracellular long Aβ species, in cell-based and in vivo studies of AD model mice.	2017	Tagami et al [109]
	Cerebral organoids; Cdk5; Isogenic; Tauopathy	Significant reduction of phosphorylated tau and its seeding activity in the brain of double transgenic mice compared with the P301S mice. Synaptic loss and impaired LTP at hippocampal CA3 region of P301S mice were attenuated by blocking p25 generation. Blockade of p25 generation reduced levels of phosphorylated tau and increased expression of synaptophysin.	2017	Seo et al [102]
	3D organoids; Aβ; Tau; AD-GWAS; iMGLs	iMGLs develop in vitro similarly to microglia in vivo, and whole-transcriptome analysis demonstrates that they are highly similar to cultured adult and fetal human microglia.	2017	Abud et al [1]
	Aβ42/Aβ40 ratio; Alzheimerogen; Aβ; Aβ Herbicides; HCE; Triazines	Neurons derived from iPSCs obtained from a FAD patient (AβPP K724 N) produced more Aβ42 versus Aβ40 than neurons derived from healthy controls iPSCs (AβPP WT). Triazines enhanced Aβ42 production in both control and AD iPSCs-derived neurons. Triazines also shifted the cleavage pattern of alcadeinα, another γ-secretase substrate, suggesting a direct effect of triazines on γ-secretase activity.	2016	Portelius et al [91]
	BACE; Neuregulin; Amyloid	Subcellular compartmentalization allows BACE1 to cleave APP in the endosomal compartment and other non-amyloid substrates in non-endosomal compartments.	2016	Ben et al [9]
	Signaling events	Over a timeframe that mirrors human development, these progenitors give rise to functional lower and upper layer neurons.	2016	Saurat et al [97]
	Fibroblasts; Postmortem; Centenarian donors	The expression of molecules that play critical roles in late-onset neurodegenerative diseases by neurons differentiated from the centenarian-iPSCs was compared to that of neurons differentiated from iPSCs derived from FAD and familial Parkinson’s disease patients.	2012	Yagi et al [132]
	DS; Development of AD pathologies	Hyperphosphorylated tau protein, a pathological hallmark of AD, was found to be localized to cell bodies and dendrites in iPSCs-derived cortical neurons from Down syndrome patients, recapitulating later stages of the AD pathogenic process.	2012	Shi et al [104]
SPORADIC AD	Aβ; Mitochondria	Neuronal cultures from some patients produced more reactive oxygen species and displayed higher levels of DNA damage. Patient-derived cells showed increased levels of oxidative phosphorylation chain complexes, whereas mitochondrial fission and fusion proteins were not affected.	2018	Birnbaum et al [13]
	PBMC; Homozygous APOE4 AD: ASUi003-A; non-demented control: ASUi004-A	hiPSCs maintained their original genotype, expressed pluripotency markers, exhibited a normal karyotype, and retained the ability to differentiate into cells representative of the three germ layers.	2017	Brookhouser et al [14]
	PBMCs;Homozygous APOE4 risk allele AD: ASUi001-A; non-demented control: ASUi002-A	hiPSCs maintained their original genotype, expressed pluripotency markers, exhibited a normal karyotype, and demonstrated the ability to differentiate into cells representative of the three germ layers.	2017	Brookhouser et al [15]
	Glucose MetS/T2DM; NMDA receptor	Redox-mediated posttranslational modification of brain proteins link Aβ and hyperglycaemia to cognitive dysfunction in MetS/T2DM and AD.	2016	Akhtar et al [2]
	Neurodegeneration; Pathology propagation; Tau oligomer seeds	Tau oligomers, but not monomers, induce accumulation of pathological, hyperphosphorylated tau. This effect was accompanied with neurite degeneration, loss of synapses, aberrant calcium homeostasis, imbalanced neurotransmitter release, and ultimately with neuronal death.	2015	Usenovic et al [124]
	SORL1 gene	The variation in SORL1 expression induction by BDNF is modulated by common genetic variants and can explain how genetic variation in this one locus can contribute to an individual’s risk of developing SAD.	2015	Young et al [136]
	Dermal fibroblasts; 82 year old female	The expression of p-tau and GSK3B, a physiological kinase of tau, in neuronal cells derived from AD-iPSCs. Treatment of neuronal cells differentiated from AD-iPSCs with an inhibitor of γ-secretase resulted in the down-regulation of p-tau.	2015	Hossini et al [44]
	BFCNs; ApoE3/E4 genotypes (AD-E3/E4)	BFCNs derived from AD-E3/E4 patients showed typical AD biochemical features evidenced by increased Aβ42/Aβ40 ratios. AD-E3/E4 neurons also exhibited altered responses to treatment with γ-secretase inhibitors compared to control BFCNs or neurons derived from patients with FAD.	2014	Duan et al [28]
	Frozen non-cryoprotected tissue; Autopsy cohort	Disease-specific iPSCs can be generated from readily available, archival biobanked tissue. This allows for rapid expansion of generating iPSCs with confirmed pathology as well as allowing access to rare patient variants that have been banked.	2014	Sproul et al [117]
FAMILIAL AD AND SPORADIC AD	Aβ; GSK3B; Hyper phosphorylation; TAU pathology	Neurons from patients with FAD and patients with SAD showed increased phosphorylation of TAU protein at all investigated phosphorylation sites. Neurons derived from patients with FAD and patients with SAD exhibited higher levels of extracellular Aβ1–40 and Aβ1–42.	2017	Ochalek et al [80]
	Calcium homeostasis; Cytokine release; Lactate secretion; Mitochondrial metabolism; Oxidative stress; Aβ production	AD astrocytes manifest hallmarks of disease pathology, including increased Aβ production, altered cytokine release, and dysregulated Ca2 + homeostasis.	2017	Oksanen et al [82]
	LC-MS/MS; Biomarker; Proteomics	Alpha-1-acid glycoprotein (ORM1) was decreased in the culture media of AD-iPSCs-derived neurons, consistent with previous observations for AD patient cerebrospinal fluid, thus validating our new strategy.	2017	Shirotani et al [113]
	hiPSC-derived astrocyte model	Chemically defined and highly efficient model provides > 95% homogeneous populations of human astrocytes within 30 days of differentiation from cortical NPCs.	2017	Jones et al [51]
	Aβ; Neurotoxicity; PS1-A246E mutation	iPSCs lines were differentiated into neuronal precursors (iPSCs-NPCs) and neurons that were subjected to Aβ toxicity assays. Neurons derived from the FAD patient have a higher susceptibility to Aβ1–42 oligomers compared with neurons coming from healthy and sAD individuals.	2017	Armijo et al [5]
	Neuroprotective activity; Apigenin	The iPSCs-derived AD neurons demonstrated a hyper-excitable calcium signaling phenotype, elevated levels of nitrite, increased cytotoxicity and apoptosis, reduced neurite length and increased susceptibility to inflammatory stress challenge from activated murine microglia, in comparison to control neurons.	2016	Balez et al [8]
	Cellular model; Synaptotoxic effects of Aβ	Upon long-term cultivation, purified cells differentiated into mature neurons exhibiting the generation of action potentials and excitatory glutamatergic and inhibitory GABAergic synapses. Most interestingly, these iPSCs-derived human neurons were strongly susceptible to the synaptotoxic actions of Aβ.	2015	Nieweg et al [79]
	Neurotoxicity; Non-toxic mutants of Aβ42;	The non-toxic mutants of Aβ42 without the “toxic” turn could prevent the propagation process of the toxic conformer of Aβ42 resulting in suppression of the formation of the toxic oligomers.	2013	Izuo et al [49]
	Disease model	Aβ oligomers accumulated in iPSCs-derived neurons and astrocytes in cells from patients with a familial APP-E693Δ mutation and SAD, leading to endoplasmic reticulum (ER) and oxidative stress.	2013	Kondo et al [55]
	Duplication of the Aβ APP (Dp)	Direct relationship between APP proteolytic processing, but not Aβ, in GSK-3β activation and tau phosphorylation in human neurons. Neurons with the genome of one sAD patient exhibited the phenotypes seen in familial AD samples.	2012	Israel et al [48]
	Drug evaluation; Preclinical; Tissue therapy; PD; DM; DMD	Each iPSCs line exhibited an intense alkaline phosphatase activity, expression of pluripotent markers, and the potential to differentiate into all three embryonic germ layers.	2012	Jang et al [50]
DOES NOT SPECIFY	Drug screening; Parkinsonism; Tauopathies; Triple MAPT-mutant	Mutant neurons expressed pathogenic 4R and phosphorylated TAU, endogenously triggered TAU aggregation, and had increased electrophysiological activity.	2018	García-León et al [37]
	DS; Hsa21 trisomy; Aβ; Cortical neurogenesis; Tau phosphorylation	Cortical neuronal differentiation shows that an increased APP gene dosage is responsible for increased β-amyloid production, altered Aβ42/40 ratio, and deposition of the pyroglutamate (E3)-containing amyloid aggregates, but not for several tau-related AD phenotypes or increased apoptosis.	2018	Ovchinnikov [85] et al
	3D culture; Bioinformatics; Proteomic	Similar analysis of post-mortem AD brain tissue revealed significant alteration in proteins involved in oxidative stress, neuro-inflammation, along with proteins related to axonal injury.	2018	Chen et al [21]
	Leptomeningeal cell; Postmortem	Leptomeningeal-derived hiPSCs lines can be generated from fresh and frozen leptomeninges, are pluripotent, and retain the karyotype of the starting cell population.	2018	Rose et al [96]
	SORL1 expression; Null, one, or two copies of the APOE4 allele	Reduced SORL1 expression only in NSCs of a patient carrying two copies of APOE4 allele with increased Aβ/SORL1 localization along the degenerated neurites. SORL1 binding to APP was largely compromised; this could be almost completely reversed by γ-secretase (but not β-secretase) inhibitor treatment.	2017	Zollo et al [142]
	PD; Cortical neurons; Macrophage; Microglia; Neuroinflammation	Co-cultures retain neuronal maturity and functionality for many weeks. Co-culture microglia express key microglia-specific markers and neurodegenerative disease-relevant genes, develop highly dynamic ramifications, and are phagocytic. Upon activation, they become more amoeboid, releasing multiple microglia-relevant cytokines.	2017	Haenseler et al [42]
	AICD; APP; CTF; Aβ;p3 peptide	The 42:40 ratio was highest for Aβ’, followed by Aβ and then p3. Mass spectrometry analysis of APP intracellular domains revealed differential processing of APP-C83, APP-C89, and APP-C99 by γ-secretase already at the ε-cleavage stage.	2017	Siegel et al [114]
	PBMCs; Yamanaka factors	The transgene-free iPSCs line showed pluripotency verified by immunofluorescent staining for pluripotency markers, and the iPSCs line was able to differentiate into the 3 germ layers in vivo. The iPSCs line also showed normal karyotype.	2017	Zhang et al [140]
	Down syndrome; Aβ; Ivermectin; Phenotypic screening; Selamectin	The authors identified the avermectins, commonly used as anthelmintic, as compounds that increase the relative production of short Aβ peptides at the expense of longer, potentially more toxic peptides. Further studies demonstrated that this effect is not due to an interaction with the core γ-secretase responsible for Aβ production.	2017	Brownjohn et al [16]
	APP; Aβ42; BACE2; Aβ DS; ETS2; RCAN1; TMED10	In vitro generated DS neural cells have abnormal metabolism of Aβ manifested by increased secretion and accumulation of Aβ granules of Aβ42 pathological isoform with upregulated expression of the APP gene.	2017	Dashinimaev et al [23]
	hTFAM; Oxidative stress; mtDNA	Expression of hTFAM significantly improved cognitive function, reducing accumulation of both 8-oxoguanine, an oxidized form of guanine, in mtDNA and intracellular Aβ in 3xTg-AD mice and increasing expression of transthyretin, known to inhibit Aβ aggregation.	2016	Oka et al [81]
	Gene-corrected version; Substituting mutation with wild-type sequence A79V mutation in PSEN1	The reported A79V-GC-iPSCs line is a very useful resource in combination with the A79V-iPSCs line in order to study pathological cellular phenotypes related to this particular mutation.	2016	Pires et al [88]
	HLC; Gene CR1	The iPSCs retained the CR1 CNV, and comparative transcriptome analyses with the human ESCs line H1 revealed a Pearson correlation of 0.956 for AD1-CR10 and 0.908 for AD1-CR14.	2016	Schroter et al [100]
	Episomal plasmids; HLC; Missense mutation TREM2	Human lymphoblast cells from a female patient possessing the mis-sense mutation TREM2 p.R47H were used to generate integration-free iPSCs employing episomal plasmids expressing OCT4, SOX2, NANOG, LIN28, c-MYC and L-MYC.	2016	Schroter et al [98]
	PBMC; Memory deficit	Integration-free CytoTune-iPS Sendai Reprogramming factors were introduced to PBMC to convert them to iPSCs without retention of virus. Three germ layer differentiation was induced to demonstrate the pluripotency of these iPSCs.	2016	Lee et al [59]
	Disease model; Human lymphoblast cells; TREM2 p.R47H variant	Human lymphoblast cells from a male patient expressing the TREM2p.R47H variant were used to generate integration-free iPS cells employing episomalplasmids expressing OCT4, SOX2, NANOG, LIN28, c-MYC and L-MY	2016	Schroter et al [99]
	Apoptosis; Kinase inhibitors; TRAIL	Wortmannin resulted in disappearance of phosphorylated AKT and activation of the main effector caspase-3 in iPSCs. These results clearly demonstrate for the first time that PI3K-AKT represents a highly essential survival-signaling pathway in iPSCs.	2016	Hossini et al [45]
	DS; MSC; Amniotic fluid	DS-iPSC derived neural cells can serve as an ideal cellular model of DS and AD and have potential for high-throughput screening of candidate drugs. Bdph may benefit DS or AD treatment by scavenging Aβ aggregates and NFTs.	2015	Chang et al [19]
	3D culture; Mechanotransduction	3D in vitro model has higher resemblance to the AD pathology than conventional 2D cultures and could be used in further studies of the disease.	2014	Zhang et al [139]
	Aβ; Glutamatergic; In vitro model	Administration of such Aβ oligomers yielded signs of the disease, including cell culture age-dependent binding of Aβ and cell death in the glutamatergic populations. Aβ-induced toxicity was selective for glutamatergic rather than GABAeric neurons present in our cultures.	2014	Vazin et al [125]
	GCSF; Aβ-induced	Demonstrate an improvement of memory and neurobehavioral function with GCSF in Aβ-induced AD model in rats.	2013	Prakash et al [92]
	Drug evaluation; Forebrain marker; Neocortical markers	The iPSCs cell-derived neuronal cells also expressed APP, β-secretase, and γ-secretase components, and were capable of secreting Aβ into the conditioned media. Aβ production was inhibited by β-secretase inhibitor, γ-secretase inhibitor (GSI), and an NSAID; however, there were different susceptibilities to all three drugs between early and late differentiation stages.	2011	Yahata et al [133]

One of the first studies involving iPSCs generated for AD were carried out in 2011 by Yahata et al. [133] and Yaqi et al. [131]. Yahata et al., [133] successfully generated forebrain neurons from human iPSCs cells, and showed that Aβ production in neuronal cells was detectable and inhibited by some typical secretase inhibitors and modulators. According to the authors, hiPSCs cell-derived neuronal cells express functional β- and γ-secretases involved in Aβ production. However, anti-Aβ drug screening using these hiPS cell-derived neuronal cells requires sufficient neuronal differentiation. Also, Yaqi et al. [131] generated iPSCs from fibroblasts of FAD patients with mutations in PS1 (A246E) and PS2 (N141I) and characterized the differentiation of these cells into neurons. The authors demonstrated that patient-derived differentiated neurons have increased Ab42 secretion, recapitulating the pathological mechanism of FAD with PS1 and PS2 mutations.

Taken together, along with others, these two studies represent, thus, critical first steps in assessing the potential of AD iPSCs to model AD. iPSCs are a pre-clinical tool for screening therapeutic compounds.

In addition, this approach is central to toxicity and efficacy testing, in the new drug development landscape and the precise engineering of the genome and transcriptional and proteomic analyses. Thus, human cells can demonstrate pathogenic mutations in vitro, which can then be functionally validated and downstream targets can be confirmed. In the future, these models can give rise to a new preclinical model for drug discovery and even personalized therapeutics based on individual’s genetics [94].

## Future directions

With the advancement of research using the iPSCs, the customization of the treatment for patients with AD is possible, reaching new insights associated with the pathogenesis and discovery of new drugs for the treatment and/or prevention, which is economically impractical at present [83]. The treatment is individualized based on the behavior of the cellular model for possibly defining the AD subgroups [83]. At present, we can model in vitro diseases to allow patient-specific therapies from newly derived AD-iPSCs to be used by considering the appropriate characterization of AD patient groups through genetic profiles and biomarkers [83].

In addition, iPSCs have some limitations. One can consider the immature and fetal population of neurons that are obtained from the iPSCs that model the AD in case of an illness of the aging [69, 73, 118, 127]. Therefore, there is a possibility to express a mutant form of LMNA, which is known to cause premature aging [46].

The challenges in the enhancement of iPSCs are directly related to modeling protocols. In iPSCs, we can highlight the level of maturity of the neurons, lack of efficient protocols to generate microglia, and few protocols of 3D differentiation that appropriately mimic the in vivo environment of the brain [107]. Now, to generate and differente iPSCs are still a time-consuming and expensive processes; however, with the improvement and development of new protocols, iPSCs can be used from an individual to direct their appropriate treatment through personalized medicine, improving the patient’s life [46].

During the formation of iPSCs, there are a concern about introducing harmful mutations. Therefore, new genome editing techniques, such as the clustered regularly interspaced short palindromic repeats (CRISPR)-associated protein 9 (Cas9) nuclease system, reduce the risk of introduction and spread of undesired mutations [134, 110]. Paquet et al. [86] accurately and efficiently generated both homozygous and heterozygous dominant AD-causing mutations using CRISPR/Cas9 [110].

Essentially, neurodifferentiated cells from iPSCs exhibit the pathological characteristics of an individual with AD in less than two months, demonstrating that cultured cells are more susceptible to display the disease characteristics than those that occur in a patient’s brain. Moreover, it is not known whether one or two differentiated cell types from iPSCs may represent the complicated disease phenotype [134].

Another limitation of iPSCs is the fact that they represent models in two dimensions, thus lacking cellular diversity, having structural complexity, and presenting physical architecture in vivo. Therefore, a fundamental approach for the development of a physiologically relevant model is to make a three-dimensional (3D) model of the neurons and glia [4, 32], revealing heterogeneous and naturally organized cellular models [4]. Normal cortical folding [66], microcephaly [56], and lissencephaly [11] are some successful organoids used to model neurodevelopmental processes and diseases [4]. Neurodegenerative disease models are still scarce; they can give new insight to model AD [4]. According to our literature survey, some authors are already using organoids in AD research, which originate from iPSCs [1, 102, 116, 117]. Therefore, defined radial glial cells can be obtained using these organoids; these cells are crucial in brain development and function, as well as associated with the organization and morphology similar to the developing human cortex [4, 93]. This model has made considerable contributions over time. Thus, supporting cells that develop along the first neurons can be crucial in modeling the onset and progression of the disease [4, 95].

Along with the progressive neurodegeneration of patients with AD, memory and the ability to learn and perform daily activities are also impaired over time. In an aging society is necessary and urgent to develop an efficient drug to treat AD, thus clinical AD may need to be reclassified into different subtypes, and the prediction of drug responsiveness may be possible based on the different subtypes [55, 47].

Considering that therapies for AD are mostly palliative, a great deal of effort is made by the scientific community to discover a drug; however, several promising candidate drugs have failed in recent clinical trials [20]. Semagacestat, for example, is a potential nontransitional state analog of γ-secretase inhibitor (GSI). All GSI studies for AD, including Semagacestat, were unsuccessful [112, 109]. Arguments against the efficacy of reducing Aβ levels in the brain are based on the results obtained while aiming a therapy for AD [12, 33, 53, 109]; the expected effect was exactly the opposite, considering that Semagacestat and another potential GSI Avagacestat worsened the cognitive decline [22, 26, 109].

Recently, quantifying small residual peptides, Tagami et al. [109], addressed the effects of Semagacestat on PS/g-secretase activity, generated during sequential cleavages upon Aβ production. The authors demonstrated seemingly contradictory actions of Semagacestat, by decreasing levels of extracellular Aβ and intracellular amyloid protein precursor intracellular cytoplasmic domain along with increased bAPP-C-terminal fragment stubs. These Semagacestat effects are clearly different from those caused by a loss of functional PSs. According to the authors, Semagacestat is a pseudo-GSI and may inhibit the liberation of product peptides by g-secretase (g-byproducts) from the membrane to the soluble space. This allows g-byproducts to accumulate in living cells. A comprehensive assessment related to g-secretase activity will allow the discovery of clinical application of g-secretase-modulating compounds [109].

From AD diagnosis, an individual has four to five years of life span. Neuroreplaced therapies will not compensate for the neuronal loss but may be used to improve existing circuits temporarily, contributing to cognitive function and quality of life [30]. Cell replacement therapy has been the most challenging because of the multifactorial nature of AD. Earlier studies in animal models with AD have shown that the transplantation of neural stem cells can improve cognition, reduce neuronal loss, and increase synaptic plasticity. This is probably because of the mechanisms that are involved in neuroprotection and trophic support rather than those involved in neuronal substitution [20].

Future studies with iPSCs need to define the cell type and which cell type is impacted in a disease phenotype [24, 35, 40]. If the genetic identity of a natural cell is defined, it is possible to correlate with the modified cell in vitro. Similar studies are already proposed regarding the retina [105, 75].

With the increased genetic information, it becomes a growing priority to translate these genotypes into their functional biological results. The formation of subtypes of neurons can contribute to the evaluation of variants within discrete cell populations, defining specific genetic contributions to disease within each cell class [107].

The involvement of the cell type in the disease, with the possibility of modulating a specific gene expression profile, will help monitor the effect on downstream pathway members and consequently allow the modification of the pathways associated with the disease by modifying the proposed disease-relevant pathways. The evaluation of the phenotypic results of these alterations will show the biological effects of the gene expression, classifying whether the gene is relevant to the disease or not [108].

The prevalence of AD is higher in women, but in men the congenital decline is more severe and early [57, 110]. Hormonal and metabolic differences in the brain may explain these distinctions between the sexes [138, 110]. The study on the sexual dimorphism of microglia phenotype, for example, in the cortex and cerebellum, has strengthened [106, 111, 103, 7]. According to Streit et al. [119], microglia may be involved through the “microglia dysfunction hypothesis”. Therefore, to elucidate the influence of sex and its contribution to neuroinflammation in the AD, future studies may include endogenous microglia and inflammation as a phenotype in chimeric models [110].

Goldstein et al. [39] pointed some suggestions for future research, such as working in isogenic systems, which are described by Woodruff et al. [129], considering the known genome variability and human physiology. In addition, working on cell -nes that have been completely sequenced and determining a true diploid sequence to the level of the genome described to date have been suggested [63, 39]. Because of the complex nature of the pathophysiology of AD, a multimodal approach may be necessary, incorporating the pharmacological segmentation of the pathology, stimulation of endogenous neurogenesis and synaptogenesis, and exogenous neuroreplacement [30].

Regarding sporadic AD, a greater challenge exists to elucidate the factors that result in the disease. It is known that there is a hereditary component and that each individual with its unique genetic background has variants that may predispose or protect for the disease. Therefore, the research seeks to discover the genetic contribution to the AD if there are phenotypic consequences in an individual with a genetic background that contains genetic variants of risk [137]. In this manner, possible paths can be disclosed, and in the future may be used to determine the factors that cause AD and to test new possible therapeutics strategies [137]. Yang et al. [134] highlighted that an appropriate control group needs to be selected; iPSCs derived from healthy individuals or family members may have totally different genetic background compared with those from individuals with AD [134].

Valuable findings have already been obtained regarding the development of AD; however, several studies still need to elucidate its effects. For example, new information related to genetic variants in individual genomes and their influence on the neuronal phenotype will facilitate to identify the chance to developing AD through the identification of molecular and biochemical phenotypes caused by these genetic variants [137]. This 3D approach can reveal the connection between neurons and glia, and these genetic factors can be advantageous to drugs discovery of when the supporting cells are crucial, or the route of interest is unknown [4, 32].

## Conclusion

The AD field of research has received valuable contributions from iPSC models. Considering that the iPSC technique is relatively recent, discovered in 2006, it is important to recognize associated advances obtained so far in AD research. The possibility to study neurons from a patient with AD in a culture dish allows observation of relevant cellular phenotypes and behaviors, following the earliest events in dementia. These research models have allowed the observation of direct molecular effects of FAD mutations and genetic risk variants, which may lead to more efficacious treatments targeting this devastating disorder.

## Abbreviations

AD: Alzheimer’s disease
ApoE4: Apolipoprotein-E4
APP: Amyloid precursor protein
ASM: Acid sphingomyelinase
Aβ: Amyloid-beta
BFCNs: Basal forebrain cholinergic neurons
Cdk5: Cyclin-dependent kinase 5
CNS: Central Nervous System
CTF: C-terminal fragment
DIAN: Dominantly Inherited Alzheimer Network
DM: Diabetes mellitus
DMD: Duchenne type muscular dystrophy
ESCs: Embryonic stem cells
FAD: Familial AD
GCSF: Granulocyte colony stimulating factor
GSI: γ-secretase inhibitors
GSM: γ-secretase modulator
HCE: Human chemical exposome
HLC: Human lymphoblast cells
hTFAM: Human mitochondrial transcriptional factor A
IMGLs: Human microglial-like cells
iPSCs: Induced pluripotent stem cells
MSC: Mesenchimal stem cells
mtDNA: Mitochondrial DNA
NFTs: Neurofibrillary tangles.
non-TSA: Non-transition state analog
NPCs: Neural progenitor cells
PBMCs: Peripheral blood mononuclear cells
PD: Parkinson’s disease
PS1: Presenilin-1
PS2: Presenilin-2
SAD: Sporadic AD
sAPPα: Soluble amyloid precursor protein-alpha
